# Beyond the fish-*Daphnia* paradigm: testing the potential for pygmy backswimmers (*Neoplea striola*) to cause trophic cascades in subtropical ponds

**DOI:** 10.7717/peerj.14094

**Published:** 2022-09-28

**Authors:** Chase J. Rakowski, Mathew A. Leibold

**Affiliations:** 1 Department of Integrative Biology, University of Texas, Austin, United States of America; 2 Department of Biology, University of Florida, Gainesville, United States of America

**Keywords:** Pleidae, Hemiptera, Copepod, Food chain, Food web, Freshwater ecology, Invertebrate predator, Plankton, Zooplankton, Phytoplankton

## Abstract

Trophic cascades, or indirect effects of predators on non-adjacent lower trophic levels, are a classic phenomenon in ecology, and are thought to be strongest in aquatic ecosystems. Most research on freshwater trophic cascades focused on temperate lakes, where fish are present and where *Daphnia* frequently dominate the zooplankton community. These studies identified that *Daphnia* often play a key role in facilitating trophic cascades by linking fish to algae with strong food web interactions. However, *Daphnia* are rare or absent in most tropical and subtropical lowland freshwaters, and fish are absent from small and temporary water bodies, where invertebrates fill the role of top predator. While invertebrate predators are ubiquitous in freshwater systems, most have received little attention in food web research. Therefore, we aimed to test whether trophic cascades are possible in small warmwater ponds where *Daphnia* are absent and small invertebrates are the top predators. We collected naturally occurring plankton communities from small fishless water bodies in central Texas and propagated them in replicate pond mesocosms. We removed zooplankton from some mesocosms, left the plankton community intact in others, and added one of two densities of the predaceous insect *Neoplea striola* to others. Following an incubation period, we then compared biomasses of plankton groups to assess food web effects between the trophic levels, including whether *Neoplea* caused a trophic cascade by reducing zooplankton. The zooplankton community became dominated by copepods which prefer large phytoplankton and exhibit a fast escape response. Perhaps due to these qualities of the copepods and perhaps due to other reasons such as high turbidity impairing predation, no evidence for food web effects were found other than somewhat weak evidence for zooplankton reducing large phytoplankton. More research is needed to understand the behavior and ecology of *Neoplea*, but trophic cascades may generally be weak or absent in fishless low latitude lowland water bodies where *Daphnia* are rare.

## Introduction

An extensive body of literature has demonstrated the importance of food web effects, including indirect top-down food web effects, or trophic cascades, with much of this work focusing on freshwater ecosystems from an early time (Ripple et al., 2016). Many of the studies on trophic cascades in freshwater pelagic ecosystems have focused on large-bodied cladocerans, especially *Daphnia*, as the herbivorous prey linking predators to autotrophs (*e.g.*, Carpenter et al., 2001; Walsh, Lathrop & Vander Zanden, 2017). The paradigm that developed from this research is that trophic cascades are common in lakes and ponds, and operate via a fish-*Daphnia*-phytoplankton food chain (Pujoni et al., 2016). However, a relative paucity of such studies were performed in tropical and subtropical lowlands, where *Daphnia* is rare (Dumont, 1994). This subset of studies suggests that the fish-*Daphnia*-phytoplankton cascade model does not apply well in warm climates, due to the lack of large herbivorous zooplankton and other factors including increased omnivory by fish and increased abundance of invertebrate predators (Rejas et al., 2005; Pujoni et al., 2016; Lacerot et al., 2021). As a result, the effects of predator removal and addition seem to be more variable in (sub)tropical lakes and ponds, and general predictions remain elusive (Rejas et al., 2005; Pujoni et al., 2016; Mayer, 2020).

Another bias in freshwater trophic cascade research, including both temperate and tropical studies, is a focus on the effects of fish rather than invertebrate predators. This bias is notable because fish tend to be absent from small and temporary water bodies whereas invertebrate predators are present in essentially all freshwater bodies. Certain invertebrate taxa such as *Bythotrephes*, *Chaoborus*, and Notonectidae have received attention, with studies showing they can have important effects on both traits and biomass of lower trophic levels (Carpenter et al., 1992; Carpenter et al., 2001; Walsh, Lathrop & Vander Zanden, 2017; Ingram & Burns, 2018; Horppila et al., 2019). However, other studies have found that invertebrate predators have weak to nonexistent top-down effects, and the food web effects of many common and widespread invertebrate predators remain unknown (*e.g.*, Mermillod-Blondin et al., 2020). Characterizing the effects of these understudied predaceous invertebrate species is important for understanding their role in ecosystems and for conservation planning, as predators and freshwater species are at a higher extinction risk than species in lower trophic levels or other habitats (Purvis et al., 2000; Strayer & Dudgeon, 2010).

Members of the family Pleidae are small but common heteropteran insects closely related to the family Notonectidae which includes *Notonecta* (backswimmers). Pleids have been much less studied than their larger cousins the notonectids, especially *Notonecta* and *Buenoa*. In this paper we specifically study the pleid *Neoplea striola* (hereafter, *Neoplea*) as our manipulated zooplanktivorous predator. *Neoplea* is a widespread inhabitant of lentic freshwater in Central and North America, and is known to tolerate low oxygen conditions (Gittelman, 1975). It is a small insect, with adults measuring 1.5 mm in length. *Neoplea* is an active hunter which uses sight, vibrations, and possibly chemicals to sense its prey (Papacek, 2001). They have been shown to attack and consume small zooplankton such as rotifers, mesozooplankton such as *Daphnia*, and even prey as large as dipteran larvae (Gittelman, 1974; Hampton & Gilbert, 2001; Papacek, 2001). However, the effects of *Neoplea* on pond communities are still not fully understood, including whether they can cause trophic cascades.

Here we report the results of a field mesocosm experiment in which we manipulated densities of *Neoplea* and zooplankton to test the effects of both of these trophic groups on plankton composition and biomass, including direct effects on the next trophic level and indirect (trophic cascade) effects of *Neoplea* on phytoplankton. Our plankton communities were composed of freshwater plankton collected locally in central Texas, with no *Daphnia* or other large-bodied cladocerans present. Due to the fast escape response of copepods, we expected *Neoplea* would primarily affect other zooplankton, especially cladocerans and ostracods (O’Brien, 1979). We further predicted that zooplankton would reduce the biovolume of total phytoplankton, as phytoplankton is the primary food source for the zooplankton in this system, and because this aligns with most freshwater pelagic trophic cascade research including some of the limited (sub)tropical literature (Menezes, Attayde & Rivera Vasconcelos, 2010; Mayer, 2020). Following logically from these first two predictions, we predicted that *Neoplea* would cause a trophic cascade, *i.e.*, indirectly increase total phytoplankton biovolume via its suppression of herbivorous zooplankton. We expected a low density of *Neoplea* to have weaker effects, leading to plankton densities intermediate between no *Neoplea* and a higher density of *Neoplea*. However, we found no evidence for food web effects of *Neoplea* and only weak evidence for effects of zooplankton on phytoplankton in the experiment. This study helps to fill a gap in knowledge about the top-down effects of invertebrate predators in freshwater bodies with warm climates.

## Materials & Methods

### Organism collection

To create a phytoplankton inoculum, we transferred phytoplankton-rich water from small fishless water bodies to two plastic tanks at the University of Texas’ Brackenridge Field Laboratory in Austin, TX, and allowed succession to proceed in the tanks for ∼3 months. We then mixed 10 L from each tank with 12 L carbonated mineral water to narcotize any zooplankton, filtered this mixture through 45 µm mesh to remove the zooplankton, and mixed well. To create a zooplankton inoculum, we collected and mixed water from several of the fishless water bodies and concentrated the mixture with a 45 µm mesh. We collected *Neoplea* from other fishless water bodies in Austin, TX.

### Experiment setup and design

We maintained 20 pond communities in 300 L round plastic tanks in an unshaded field at Brackenridge Field Laboratory. We covered the tanks in one mm^2^ screens to prevent insect immigration, filled the tanks with CaCO_3_-rich well water and maintained constant water depths using float valves. No sediment was added. Prior to the experiment, we analyzed total N and P in the water following standard American Public Health Association methods (APHA, 1989). We then added NaNO_3_ to bring total N to 14 mg/L N and added NaH_2_PO_4_ ⋅H_2_O to bring total P to 1.55 mg/L P. These are the total N and P concentrations in COMBO medium, a eutrophic medium commonly used for culturing plankton (Kilham et al., 1998). Every five or six days thereafter for six weeks, enough of both nutrients were added to each tank to compensate for a 5% daily loss rate from the water column (as per Hall et al., 2004); this same amount of both nutrients was also added immediately following the first sampling (methods described below) after a 22-day pause.

We inoculated each tank with 600 mL of the phytoplankton inoculum. After allowing phytoplankton to reproduce for five days, we added 600 mL of zooplankton inoculum to 15 of the tanks. As there may have been phytoplankton and picoplankton strains in the zooplankton inoculum not represented in the phytoplankton inoculum, we added some of the filtrate left from concentrating the zooplankton inoculum to the other five tanks to ensure all tanks received the same strains. Lastly, after allowing the zooplankton to reproduce for 15 days, over the course of three days we added 40 *Neoplea* adults to five tanks with zooplankton and 80 *Neoplea* adults to another five tanks. We standardized *Neoplea* additions by the number of individuals rather than on body size, as there was little variation in the size of adult *Neoplea*. Thus there were four treatments: no zooplankton added (“no zoop.”), zooplankton but no *Neoplea* added (“no *Neoplea*”), zooplankton and 40 *Neoplea* added (“40 *Neoplea*”), and zooplankton and 80 *Neoplea* added (“80 *Neoplea*”). Each treatment was replicated five times for a total of 20 mesocosms arranged randomly.

### Sampling and biomass estimation

We sampled zooplankton and phytoplankton 40 days after adding the *Neoplea*, and then again six days later. To sample zooplankton, we used tube samplers to collect ten whole water column subsamples spread across each tank, and pooled them into a 10 L sample for each tank. We filtered this sample through 65 µm mesh, returning any predators to the tank, and preserved the retained organisms in 10% Lugol’s solution. To sample phytoplankton, we used a 1-cm diameter PVC pipe to collect three whole water column subsamples spread over each tank and pooled them into a 50 mL sample, using a different PVC pipe for each tank. We preserved these phytoplankton samples in 10% Lugol’s solution. We additionally estimated surviving *Neoplea* populations after the second sampling event by using a dipnet to count individuals until we returned three successive empty sweeps.

To estimate biomass of zooplankton taxa, we identified, counted, and measured zooplankton in subsamples such that for each taxon, we counted at least 25 individuals or 10% of the sample –whichever came first –and at least 50 total individuals. We used an ocular micrometer to measure the length of each crustacean and individual *Spirostomum* to the nearest half increment, or 0.24 mm, and to measure the length and width of each rotifer and width of *Spirostomum* to the nearest 0.05 increment, or 0.024 mm. We used length-mass regressions to convert crustacean length to dry mass (McCauley (1984) for *Scapholeberis*, Culver et al. (1985) for copepods, and Anderson, Darring & Benke (1998) for ostracods). We converted *Spirostomum* dimensions to dry mass by approximating cells as cylinders and assuming a 10:1 biovolume:dry mass ratio. When we were able to identify rotifers to species, we converted the rotifers’ dimensions to dry mass using species-specific equations from the EPA protocol (EPA, 2016). For other rotifers, we estimated their biovolume using biovolume equations and then converted to dry mass assuming the same 10:1 biovolume:dry mass ratio (McCauley, 1984).

To estimate biovolumes of phytoplankton taxa, we calculated densities of each morphospecies with a hemocytometer. We counted 50 cells or 25 nL of the most common morphospecies –whichever came first –and we counted at least 100 nL for less common taxa. We captured several micrographs of each morphospecies from various tanks and sampling dates, and used ImageJ to measure the cell dimensions of at least 15 cells for all but the rarest morphospecies (Schneider, Rasband & Eliceiri, 2012). We then calculated the biovolume of each cell using geometric approximations (Table S1).

### Data analysis

Based on previous research, we expected *Neoplea* to have differential effects on zooplankton based on behavior and size (Rakowski et al., 2021). Copepods have faster escape responses than the other zooplankton present, so we analyzed their biomass separately. Then we analyzed the sum of all other zooplankton biomass (“non-copepods”) as we predicted *Neoplea* would primarily affect these other taxa. We also separately analyzed the major groups of non-copepod zooplankton, including cladocerans and ostracods as one group (grouped for similarity in size, morphology, and slow swimming speed), *Spirostomum* as its own distinct category, and lastly rotifers. We expected zooplankton to either affect the whole phytoplankton community, or alternatively to affect only a certain size class, either large or small morphospecies. Therefore we analyzed total phytoplankton biovolume as well as the summed biovolume of larger morphospecies and of smaller morphospecies.

We tested for a difference in *Neoplea* survival between the two densities using a *t* test. To analyze the effects of zooplankton and *Neoplea* additions on the biomass of plankton groups, we fit a generalized linear mixed model in the gamma family (gamma GLMM) for each plankton grouping using the *lme4* package (Bates et al., 2015). We included tank as a random effect to account for the repeated measures, and fixed effects for zooplankton addition and initial *Neoplea* density. We also fit the nested models excluding *Neoplea* density as well as the null models with neither *Neoplea* density nor zooplankton addition, and used likelihood ratio tests to assess whether including either addition significantly improved model fit. We used one-tailed hypothesis tests when we predicted a biomass change in a particular direction. These cases included the effect of zooplankton addition on the biomass of zooplankton groups (hypothesizing a positive change) and for the effect of zooplankton and *Neoplea* addition on total phytoplankton (hypothesizing a negative and positive change, respectively). In all other cases we used two-tailed hypothesis tests since it was conceivable that a reduction of one plankton group could have benefited another plankton group in the same trophic level. For example, zooplankton could conceivably have had a negative effect on large phytoplankton and a positive effect on small phytoplankton. All analyses were performed in R v. 3.5.3 (R Core Team, 2017).

## Results

On average *Neoplea* survived at a rate of 74%, with no significant difference in survival between the two densities (d.f. = 7.98, *t* =  − 0.518, *p* = 0.618). No evidence was found of *Neoplea* reproduction during the experiment.

**Figure 1:**
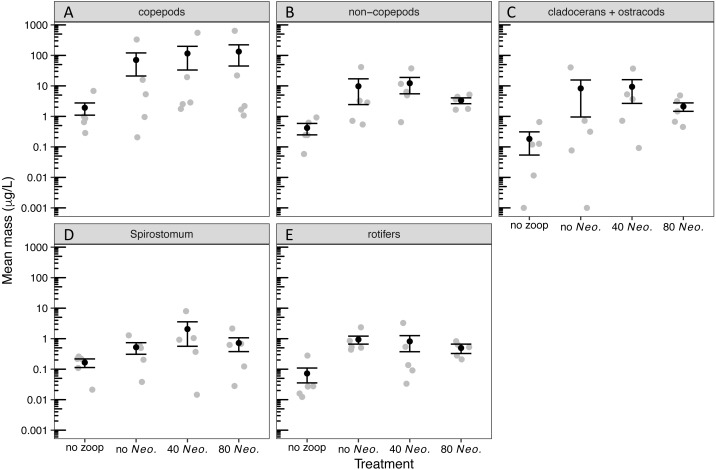
Dry mass of zooplankton groups by treatment. Black dots represent means, error bars represent ±1 standard error of the mean, and gray dots represent means of individual tanks. (A) copepods, (B) non-copepod zooplankton, (C) cladocerans and ostracods, (D) *Spirostomum*, and (E) rotifers. Note that 0.001 was added to all values to allow plotting on a log scale.

By the sampling dates, the zooplankton community had become dominated by copepods (Table S2). However, cladocerans, *Spirostomum*, and rotifers all individually composed at least 15% of the zooplankton mass on average in one or more treatments (Fig. S1). A small amount of zooplankton, mostly copepods, became established in the control tanks receiving no zooplankton (Fig. S1, Fig. 1). The zooplankton additions were successful in sustaining a significant increase in biomass of all zooplankton groups (Tables 1, 2, Fig. 1). However, *Neoplea* additions had no significant effects on the biomass of any zooplankton group (Fig. 1, Table 1). Mean copepod mass was higher and the mean proportion of rotifers was lower the more *Neoplea* were added (Fig. S1), but neither copepod nor rotifer mass was significantly affected by *Neoplea* (Fig. 1, Table 1).

**Table 1:** Results of likelihood ratio tests comparing nested GLMMs for biomass of zooplankton groups. Displayed are the degrees of freedom (Df), deviance (inverse goodness of fit), and *P* value for comparison against the model above, for biomass of (A) copepods, (B) non-copepod zooplankton, (C) cladocerans and ostracods, (D) Spirostomum, and (E) rotifers. *P* values <0.05 are bolded.

Model	Df	Deviance	*P*
**(A)** copepods			
random intercepts only (null)	3	80.450	
zooplankton addition	4	77.195	**0.036**
zooplankton + *Neoplea* addition	5	77.108	0.768
**(B)** non-copepod zooplankton			
random intercepts only (null)	3	185.62	
zooplankton addition	4	174.48	**<0.001**
zooplankton + *Neoplea* addition	5	174.47	0.466
**(C)** cladocerans and ostracods			
random intercepts only (null)	3	81.668	
zooplankton addition	4	76.539	**0.012**
zooplankton + *Neoplea* addition	5	76.106	0.511
**(D)** *Spirostomum*			
random intercepts only (null)	3	52.082	
zooplankton addition	4	49.017	**0.040**
zooplankton + *Neoplea* addition	5	48.974	0.836
**(E)** rotifers			
random intercepts only (null)	3	85.087	
zooplankton addition	4	71.911	**<0.001**
zooplankton + *Neoplea* addition	5	71.287	0.430

**Table 2:** Coefficient estimates for GLMMs analyzing the effect of zooplankton addition on the biomass of zooplankton groups. Estimates are displayed along with their standard errors (SE) and natural exponential functions [“Exp(estimate)”] for (A) copepods, (B) non-copepod zooplankton, (C) cladocerans and ostracods, (D) Spirostomum, and (E) rotifers. The natural exponential functions of estimates can be interpreted as multiplicative effects (*e.g.*, zooplankton addition resulted in a 7.66-fold increase in copepod mass). The effect of Neoplea addition is not included due to likelihood ratio tests indicating the term was not significant for any zooplankton group.

**Parameter**	**Estimate**	**SE**	**Exp(estimate)**
**(A)** copepods			
intercept	0.382	0.932	
zooplankton addition	2.036	1.077	7.660
**(B)** non-copepod zooplankton			
intercept	−1.184	0.559	
zooplankton addition	2.519	0.646	12.416
**(C)** cladocerans and ostracods			
intercept	−1.346	0.704	
zooplankton addition	2.003	0.816	7.411
**(D)** *Spirostomum*			
intercept	−1.257	0.459	
zooplankton addition	1.018	0.538	2.768
**(E)** rotifers			
intercept	−3.159	0.510	
zooplankton addition	2.572	0.588	13.092

**Figure 2:**
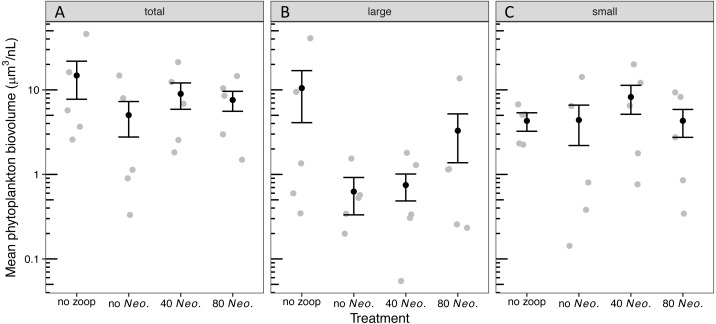
Biovolume of phytoplankton groups by treatment. Black dots represent means, error bars represent ±1 standard error of the mean, and gray dots represent means of individual tanks. (A) total phytoplankton, (B) large phytoplankton, and (C) small phytoplankton.

The phytoplankton community became dominated by ovoid single-celled green algae, with pennate diatoms contributing the next most biovolume in tanks with no zooplankton added and *Oocystis* contributing the next most biovolume in tanks with 80 *Neoplea* added (Fig. S2, Table S1). While the average total phytoplankton biovolume with zooplankton was less than half of the average without zooplankton, there was no significant effect of zooplankton addition on total phytoplankton biovolume due to large variation within treatments (Fig. S2, Fig. 2A, Tables 3A, 4A). The summed biovolume of the largest morphospecies, including the larger ovoid chlorophytes, pennate diatoms, and *Oocystis*, was 77.9% lower in the presence of zooplankton, although the difference was only marginally significant (Fig. 2B, Tables 3B, 4B). On the other hand, summed biovolume of the smaller morphospecies (small ovoid chlorophytes, *Chlorella*, *Selenastrum*, and photosynthetic picoplankton) was not significantly impacted by zooplankton addition (Fig. 2C, Tables 3C, 4C). Similarly, *Neoplea* addition had no significant effects on the biovolume of any phytoplankton grouping (Fig. 2, Table 3).

**Table 3:** Results of likelihood ratio tests comparing nested GLMMs for biovolume of phytoplankton groupings. Displayed are the degrees of freedom (Df), deviance (inverse goodness of fit), and *P* value for comparison against the model above, for biovolume of (A) total phytoplankton, (B) larger phytoplankton (large ovoid chlorophytes, Oocystis, and pennate diatoms), and (C) smaller phytoplankton (small ovoid chlorophytes, green picoplankton, *Chlorella*, and *Selenastrum*).

**Model**	**Df**	**Deviance**	***P***
**(A)** total phytoplankton			
random intercepts only (null)	3	786.53	
zooplankton addition	4	785.50	0.155
zooplankton + *Neoplea* addition	5	783.90	0.103
**(B)** larger phytoplankton			
random intercepts only (null)	3	101.79	
zooplankton addition	4	98.06	0.053
zooplankton + *Neoplea* addition	5	97.32	0.389
**(C)** smaller phytoplankton			
random intercepts only (null)	3	749.71	
zooplankton addition	4	749.25	0.500
zooplankton + *Neoplea* addition	5	748.87	0.535

**Table 4:** Coefficient estimates for GLMMs analyzing the effect of zooplankton addition on the biovolume of phytoplankton groupings. Estimates are displayed along with their standard errors (SE) and natural exponential functions [“Exp(estimate)”] for (A) total phytoplankton, (B) larger phytoplankton (large ovoid chlorophytes, Oocystis, and pennate diatoms), and (C) smaller phytoplankton (small ovoid chlorophytes, green picoplankton, *Chlorella*, and *Selenastrum*). The natural exponential functions of estimates can be interpreted as multiplicative effects (*e.g.*, zooplankton addition reduced large phytoplankton to 22.1% of the control biovolume). The effect of Neoplea addition is not included due to likelihood ratio tests indicating the term was not significant for any phytoplankton grouping.

**Parameter**	**Estimate**	**SE**	**Exp(estimate)**
**(A)** total phytoplankton			
intercept	9.000	0.594	
zooplankton addition	−0.708	0.686	0.493
**(B)** large phytoplankton			
intercept	8.147	0.650	
zooplankton addition	−1.511	0.748	0.221
**(C)** small phytoplankton			
intercept	8.269	0.654	
zooplankton addition	−0.512	0.754	0.599

## Discussion

Our results show that *Neoplea* had no demonstrable effect on the plankton in this experiment. Furthermore, the copepod-dominated zooplankton only caused a marginally significant reduction of larger phytoplankton taxa. Any effect of *Neoplea* on phytoplankton composition or biomass would have likely been mediated by an effect on zooplankton composition or biomass, as *Neoplea* are carnivorous. Therefore it is unsurprising there was no indirect effect of *Neoplea* on phytoplankton, or trophic cascade, considering there were only weak or nonexistent direct effects between the three trophic levels.

There are several potential reasons why *Neoplea* did not reduce the biomass of any zooplankton groups in our experiment. It is possible that *Neoplea* consumed chironomid larvae, which were common in the mesocosms but not effectively sampled due to their benthic nature and our sampling methods which targeted plankton. *Neoplea* has been observed to consume chironomid larvae (Papacek, 2001). Indeed, the *Neoplea* were most commonly observed clinging to the sides and bottom of the mesocosms, putting them in close proximity to the chironomids. In nature, *Neoplea* is normally found clinging to submerged vegetation (Gittelman, 1974). However, it was impossible to observe the *Neoplea* throughout most of the experiment due to the high density of phytoplankton. This high turbidity also may have made it difficult for the *Neoplea* to hunt effectively, as they can detect prey visually and are most commonly found in clear waters, though they can also detect prey by tactile and possibly chemosensory methods (Gittelman, 1974; Papacek, 2001). When the *Neoplea* did swim through the water column, they would have mostly encountered copepods, the largest and dominant group of zooplankton. However, even in clear water copepods are relatively resistant to predation due to their fast escape response. Among copepods, diaptomids have an especially fast escape response, and the diaptomid *Arctodiaptomus* dominated our mesocosms (O’Brien, 1979). Therefore it may not be surprising that the *Neoplea* were unsuccessful in suppressing the zooplankton in the experiment. While other more easily captured prey were also present, it may have been difficult for the *Neoplea* to encounter these rare prey among all the copepods in a turbid environment.

The food web ecology of *Neoplea* and pleids generally will remain unclear without further research. More behavioral research on these diminutive predators is needed to better understand their hunting habits, such as where in the habitat they make most of their captures, their relative dependence on different senses for prey detection, and their relative preference for various prey. Experimental work is needed to better quantify the ecology of pleids, and these experiments will likely benefit from better replicating their preferred habitat of clear, still water with submerged vegetation. A higher population density may be necessary for their food web effects to be apparent when they are the sole predator.

The weak effects of zooplankton on phytoplankton in the experiment may be explained by the dominance of copepods in the zooplankton community. Unlike *Daphnia*, copepods do not generally impose strong top-down control on community phytoplankton biomass, due in part to their selective grazing on larger phytoplankton (Sommer & Sommer, 2006). Indeed, the copepod-dominated zooplankton only reduced larger phytoplankton, and even this effect was marginal, fitting the general understanding of copepods’ top-down effects on phytoplankton in ecosystems with copepod-dominated herbivore communities such as open oceans (Sommer & Sommer, 2006). Such weak top-down control of crustacean zooplankton on phytoplankton may be more pervasive at low latitudes, as *Daphnia* is largely absent from tropical and subtropical lowland freshwaters and copepods are instead more likely to dominate (Dumont, 1994; Havens & Beaver, 2011).

Between the weak effect of copepods on phytoplankton biomass and the ability of copepods to evade predation much more easily than *Daphnia*, it appears likely that trophic cascades mediated by zooplankton are less common in warm, low-latitude lakes and ponds than in their colder counterparts (Rejas et al., 2005). If this is the case, then differences in plankton biomass among these warm-climate lakes and ponds may stem largely from bottom-up effects. For example, phytoplankton biomass may be largely controlled by nutrient loading and zooplankton biomass may be generally controlled by access to edible phytoplankton. Furthermore, the strategy of biomanipulation –or top predator addition to control algal blooms in eutrophic lakes –may have a low chance of success in warm climates, at least with invertebrate predators. Instead, the often more difficult method of reducing nutrient loading may be necessary to control algal blooms in fishless warm-climate ponds.

## Conclusions

We predicted that *Neoplea* would suppress non-copepod zooplankton, and that zooplankton would suppress phytoplankton, resulting in a trophic cascade where *Neoplea* indirectly increased phytoplankton biomass. Instead, *Neoplea* had no significant effect on plankton biomass or composition in this field mesocosm experiment. The zooplankton, dominated by copepods and lacking *Daphnia* as is typical of lowland tropical and subtropical lakes and ponds, only weakly reduced larger phytoplankton. While our data cannot definitively explain these weak effects, they could have resulted from *Neoplea* consuming benthic prey which was not effectively sampled, or from the dominance of copepods which are adept at evading capture and which selectively feed on larger phytoplankton. This study suggests that lentic ecosystems dominated by *Neoplea* and copepods may be characterized by weak top-down control. This represents another example of a weak or non-existent trophic cascade in lowland tropical or subtropical lentic freshwater, which appears to be more likely in these systems than in colder lakes and ponds. However, more research will be needed to achieve a clearer understanding of the ecological impacts of pleids. A better understanding of the ecology of these and other understudied freshwater invertebrate predators will be important for conservation planning, as predators and freshwater species are more threatened with extinction than lower trophic levels or terrestrial or marine species.

##  Supplemental Information

10.7717/peerj.14094/supp-1 Supplemental Information 1 Phytoplankton morphospecies/taxa and their cell shape and mean cell biovolume Taxa are listed in descending order of dominance defined by mean biovolume across all samples.

10.7717/peerj.14094/supp-2 Supplemental Information 2 Zooplankton taxa, groupings, and dry biomass across all samples Taxa (and stages) are listed in descending order of dominance, determined by median biomass or by mean biomass when the median is 0. Eight taxa not listed here, all rotifers, were rare and represented a negligible contribution to total zooplankton mass.

10.7717/peerj.14094/supp-3 Supplemental Information 3 Zooplankton composition across trophic treatments (A) Stacked bar chart of mean dry mass of zooplankton groups by treatment. (B) Stacked bar chart of mean proportions of total zooplankton dry mass each group comprises, by treatment.

10.7717/peerj.14094/supp-4 Supplemental Information 4 Phytoplankton composition across trophic treatments Stacked bar chart of mean biovolume of phytoplankton morphospecies, by treatment.

10.7717/peerj.14094/supp-5 Supplemental Information 5 Analysis of zooplankton composition and biomass R script analyzing all zooplankton data.

10.7717/peerj.14094/supp-6 Supplemental Information 6 Analysis of phytoplankton composition and biovolume R script analyzing all phytoplankton data

10.7717/peerj.14094/supp-7 Supplemental Information 7 Zooplankton biomass data Biomass of each zooplankton morphospecies in each sample. There are 20 mesocosms and two sampling dates (40 samples).

10.7717/peerj.14094/supp-8 Supplemental Information 8 Phytoplankton biovolume data Biovolume (a proxy for biomass) of each phytoplankton morphospecies in each sample. There are 20 mesocosms and two sampling dates (40 samples).

10.7717/peerj.14094/supp-9 Supplemental Information 9 All variables in both the zooplankton biomass and the phytoplankton biovolume data files

## Additional Information and Declarations

### Data Availability

The following information was supplied regarding data availability: The raw data is available in the Supplementary Files.

## References

[ref-1] Anderson DH, Darring S, Benke AC (1998). Growth of crustacean meiofauna in a forested floodplain swamp: implications for biomass turnover. Journal of the North American Benthological Society.

[ref-2] APHA (1989). Standard methods for the examination of water and wastewater.

[ref-3] Bates D, Maechler M, Bolker B, Walker S (2015). Fitting linear mixed-effects models using lme4. Journal of Statistical Software.

[ref-4] Carpenter S, Cole J, Hodgson J, Kitchell J, Pace M, Bade D, Cottingham K, Essington T, Houser J, Schindler D (2001). Trophic cascades, nutrients, and lake productivity: whole-lake experiments.

[ref-5] Carpenter S, Kraft C, Wright R, Xi H, Soranno P, Hodgson J (1992). Resilience and resistance of a lake phosphorus cycle before and after food web manipulation.

[ref-6] Culver DA, Boucherle MM, Bean DJ, Fletcher JW (1985). Biomass of freshwater crustacean zooplankton from length-weight regressions. Canadian Journal of Fisheries and Aquatic Sciences.

[ref-7] Dumont H (1994). On the diversity of Cladocera in the tropics.

[ref-8] EPA (2016). Standard operating procedure for zooplankton analysis (LG403, Revision 07).

[ref-9] Gittelman SH (1974). The habitat preference and immature stages of Neoplea striola (Hemiptera: Pleidae). Journal of the Kansas Entomological Society.

[ref-10] Gittelman SH (1975). Physical gill efficiency and winter dormancy in the pigmy backswimmer, Neoplea striola (Hemiptera: Pleidae). Annals of the Entomological Society of America.

[ref-11] Hall SR, Leibold MA, Lytle DA, Smith VH (2004). Stoichiometry and planktonic grazer composition over gradients of light, nutrients, and predation risk. Ecology.

[ref-12] Hampton SE, Gilbert JJ, Sanoamuang L, Segers H, Shiel RJ, Gulati RD (2001). Observations of insect predation on rotifers. Rotifera IX.

[ref-13] Havens KE, Beaver JR (2011). Composition, size, and biomass of zooplankton in large productive Florida lakes. Hydrobiologia.

[ref-14] Horppila J, Härkönen L, Hellén N, Estlander S, Pekcan-Hekim Z, Ojala A (2019). Rotifer communities under variable predation-turbulence combinations. Hydrobiologia.

[ref-15] Ingram T, Burns ZD (2018). Top-down control by an aquatic invertebrate predator increases with temperature but does not depend on individual behavioral type. Ecology and Evolution.

[ref-16] Kilham SS, Kreeger DA, Lynn SG, Goulden CE, Herrera L (1998). COMBO: a defined freshwater culture medium for algae and zooplankton. Hydrobiologia.

[ref-17] Lacerot G, Kosten S, Mendonça R, Jeppesen E, Attayde JL, Mazzeo N, Teixeira-de Mello F, Cabana G, Arim M, Cantarino Gomes JH, Tserenpil S, Scheffer M (2021). Large fish forage lower in the food web and food webs are more truncated in warmer climates. Hydrobiologia.

[ref-18] Mayer T (2020). Interactions of fish, algae, and abiotic factors in a shallow, tropical pond. Hydrobiologia.

[ref-19] McCauley E, Downing JA, Rigler FH (1984). The estimation of the abundance and biomass of zooplankton in samples. A manual for the assessment of secondary productivity in fresh waters.

[ref-20] Menezes RF, Attayde JL, Rivera Vasconcelos F (2010). Effects of omnivorous filter-feeding fish and nutrient enrichment on the plankton community and water transparency of a tropical reservoir. Freshwater Biology.

[ref-21] Mermillod-Blondin F, Marmonier P, Tenaille M, Lemoine DG, Lafont M, Vander Vorste R, Simon L, Volatier L (2020). Bottom-up processes control benthic macroinvertebrate communities and food web structure of fishless artificial wetlands. Aquatic Ecology.

[ref-22] O’Brien WJ (1979). The predator–prey interaction of planktivorous fish and zooplankton: recent research with planktivorous fish and their zooplankton prey shows the evolutionary thrust and parry of the predator–prey relationship. American Scientist.

[ref-23] Papacek M (2001). Small aquatic and ripicolous bugs (Heteroptera: Nepomorpha) as predators and prey: the question of economic importance. European Journal of Entomology.

[ref-24] Pujoni DGF, Maia-Barbosa PM, Barbosa FAR, Fragoso Jr CR, van Nes EH (2016). Effects of food web complexity on top-down control in tropical lakes. Ecological Modelling.

[ref-25] Purvis A, Gittleman JL, Cowlishaw G, Mace GM (2000). Predicting extinction risk in declining species. Proceedings of the Royal Society of London Series B: Biological Sciences.

[ref-26] Rakowski CJ, Farrior CE, Manning SR, Leibold MA (2021). Predator complementarity dampens variability of phytoplankton biomass in a diversity-stability trophic cascade. Ecology.

[ref-27] R Core Team (2017).

[ref-28] Rejas D, Declerck S, Auwerkerken J, Tak P, De Meester L (2005). Plankton dynamics in a tropical floodplain lake: fish, nutrients, and the relative importance of bottom-up and top-down control. Freshwater Biology.

[ref-29] Ripple WJ, Estes JA, Schmitz OJ, Constant V, Kaylor MJ, Lenz A, Motley JL, Self KE, Taylor DS, Wolf C (2016). What is a trophic cascade?. Trends in Ecology & Evolution.

[ref-30] Schneider CA, Rasband WS, Eliceiri KW (2012). NIH Image to ImageJ: 25 years of image analysis. Nature Methods.

[ref-31] Sommer U, Sommer F (2006). Cladocerans versus copepods: the cause of contrasting top–down controls on freshwater and marine phytoplankton. Oecologia.

[ref-32] Strayer DL, Dudgeon D (2010). Freshwater biodiversity conservation: recent progress and future challenges. Journal of the North American Benthological Society.

[ref-33] Walsh JR, Lathrop RC, Vander Zanden MJ (2017). Invasive invertebrate predator. Bythotrephes longimanus, reverses trophic cascade in a north-temperate lake. Limnology and Oceanography.

