# Supplementary figures and images for: Beyond the fish-Daphnia paradigm: testing the potential for pygmy backswimmers (Neoplea striola) to cause trophic cascades in subtropical ponds
Source: PeerJ. 2022 Sep 28;10:e14094. doi: 10.7717/peerj.14094 (PMC9526409; doi:10.7717/peerj.14094)

# Zooplankton group

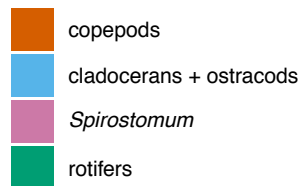

A

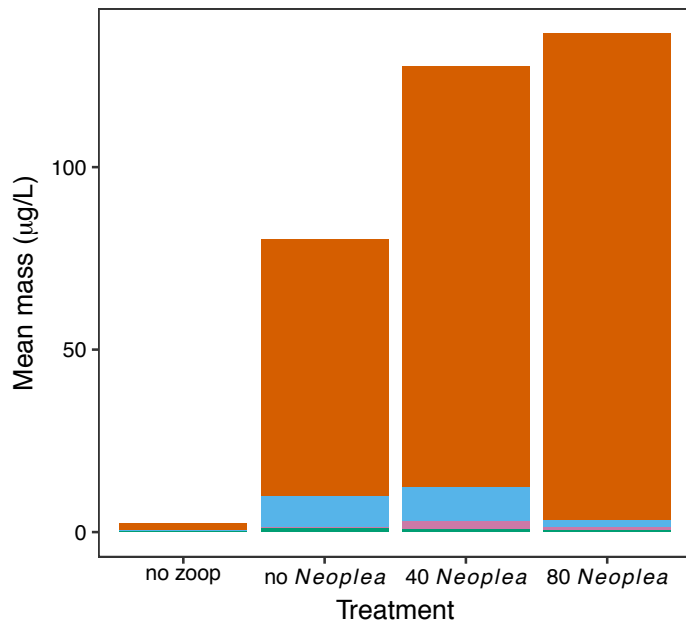

B

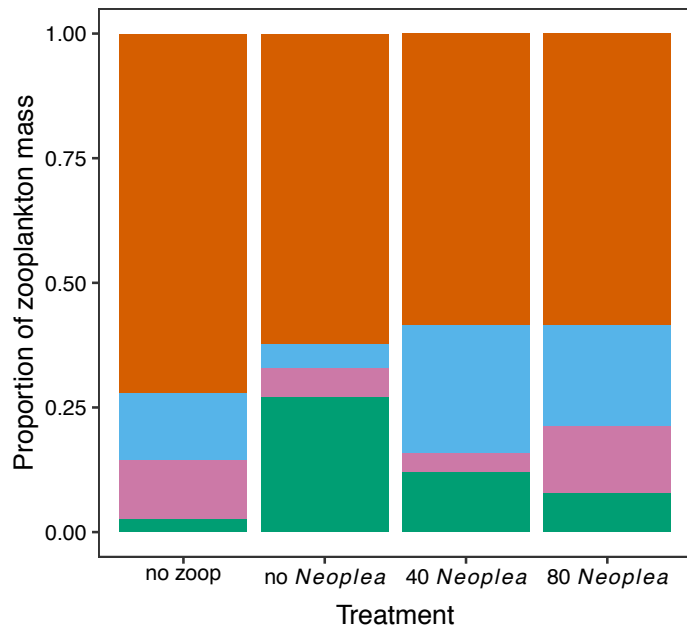

Supplement: Supplemental Information 3 — (A) Stacked bar chart of mean dry mass of zooplankton groups by treatment. (B) Stacked bar chart of mean proportions of total zooplankton dry mass each group comprises, by treatment. [file peerj-10-14094-s003.pdf]
