# Supplementary material for: Beyond the fish-Daphnia paradigm: testing the potential for pygmy backswimmers (Neoplea striola) to cause trophic cascades in subtropical ponds
Source: PeerJ. 2022 Sep 28;10:e14094. doi: 10.7717/peerj.14094 (PMC9526409; doi:10.7717/peerj.14094)
Supplement: Supplemental Information 4 — Stacked bar chart of mean biovolume of phytoplankton morphospecies, by treatment. [file peerj-10-14094-s004.pdf]

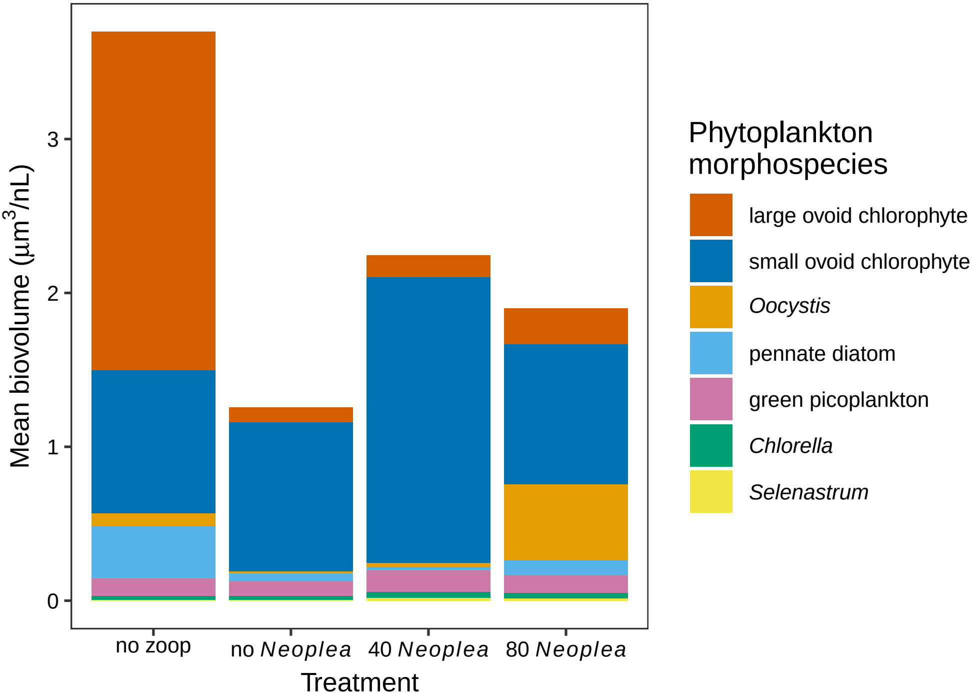

Mean biovolume (µm³/nL)
0
1
2
3
no zoop
no *Neoplea*
40 *Neoplea*
80 *Neoplea*
Treatment
Phytoplankton morphospecies
large ovoid chlorophyte
small ovoid chlorophyte
Oocystis
pennate diatom
green picoplankton
Chlorella
Selenastrum
